# Complete Genome Sequence of an African Raw Honey Bacterial Isolate, Bacillus safensis Strain AHB11

**DOI:** 10.1128/mra.00516-22

**Published:** 2022-10-26

**Authors:** Carson J. Shin, Tamara J. O’Connor

**Affiliations:** a Department of Biological Chemistry, The Johns Hopkins University School of Medicine, Baltimore, Maryland, USA; University of Rochester School of Medicine and Dentistry

## Abstract

We present the complete genome sequence of Bacillus safensis strain AHB11, which was isolated from African raw honey from Kajo Keji, South Sudan, that had been purchased from a third-party vendor. The genome is composed of a 3,697,357-bp chromosome and a 7,105-bp plasmid, collectively encompassing 3,699 predicted protein-coding sequences and 110 RNA genes.

## ANNOUNCEMENT

Raw honey has garnered much attention for its antimicrobial activity against an assortment of pathogenic bacteria (1–12). Due to its potent antimicrobial activity, honey is considered a very restrictive niche, with only a few spore-forming microorganisms being able to survive this harsh environment (13–20). Here, we report the complete genome sequence of a bacterium that was isolated from African raw honey originating in Kajo Keji, South Sudan, which we defined as Bacillus safensis and designated strain AHB11 for African honey bacterial isolate 11.

The bacterium was isolated by plating diluted African raw honey (50% [v/v] in sterile water) on *N*-(2-acetamido)-2-aminoethanesulfonic acid) (ACES)-buffered charcoal yeast extract (CYE) medium (21) containing 12 g/L agar, 0.135 g/L cysteine, 0.4 g/L iron(III) nitrate, and 0.1 mg/mL thymidine and growing the culture at 37°C for 20 h. To isolate DNA, bacteria were cultured from a single colony at 37°C for 20 h in liquid AYE medium with aeration.

DNA was extracted from bacteria using a DNeasy blood and tissue kit (Qiagen) following the manufacturer’s instructions. To lyse the bacteria, cells were incubated at 37°C for 3 h in lysis buffer (20 mM Tris-HCl [pH 8], 2 mM EDTA, 1.2% Triton X-100) supplemented with 20 mg/mL lysozyme and then were treated with proteinase K in Qiagen AL buffer at 56°C for 2 h. DNA was then quantified using a Tecan spectrophotometer equipped with a NanoQuant microplate.

The genome was sequenced and assembled at the Microbial Genome Sequencing Center (MiGS) (Pittsburgh, PA, USA). The short-read sequencing library was prepared using the Illumina DNA preparation kit, including fragmentation and size selection (320 bp). Paired-end sequencing (150 bp) was performed on an Illumina NextSeq2000 system. Read quality (quality scores of >Q30) and adapter trimming was performed with bcl2fastq v2.20.0.445 (22), generating 3,619,373 reads. The long-read sequencing library was prepared using the genomic DNA by ligation kit (Oxford Nanopore Technologies) following the manufacturer’s instructions and was sequenced with a Nanopore MinION R9.4.1 flow cell. Quality control and adapter trimming were performed using Guppy v5.0.16 (Oxford Nanopore Technologies) in high-accuracy base-calling mode and Porechop v0.2.3_seqan2.1.1 (23), generating 107,444 reads with an *N*_50_ value of 965 bp. Genome assembly and circularity determination were performed using Unicycler v0.4.8 (24), and assembly statistics were evaluated using QUAST v5.0.2 (25). For all software, default parameters were used unless otherwise indicated. The bacterial genus and species were defined based on the greatest sequence homology of 16S rRNA, 23S rRNA, *rpoB*, *gyrB*, and *recA* genes using BLAST (26).

The Bacillus safensis strain AHB11 genome consisted of a chromosome of 3,697,357 bp, with a GC content of 44.7% and 167× genome coverage, and a single plasmid of 7,105 bp. The genome was annotated by NCBI using the Prokaryotic Genome Annotation Pipeline (PGAP) (27), which identified 3,803 genes, including 3,693 coding DNA sequences (CDSs), 24 rRNAs, 81 tRNAs, 1 transfer-messenger RNA, and 4 noncoding RNAs. The same method was used to annotate the plasmid, referred to as pAHB11, which identified 6 CDSs.

### Data availability.

The complete genome sequences of Bacillus safensis strain AHB11 and its plasmid were deposited in GenBank with accession numbers CP097374 and CP097375, respectively, with BioProject accession number PRJNA836921 and BioSample accession number SAMN28178910.
